# The combination of arterial lactate level with GCS-pupils score to evaluate short term prognosis in traumatic brain injury: a retrospective study

**DOI:** 10.1186/s12883-022-02970-1

**Published:** 2022-11-15

**Authors:** Yu-Mei Wang, Ning Zhu, Yi-Min Zhou, Rui Su, Hong-Liang Li, Jian-Xin Zhou

**Affiliations:** Department of Critical Care Unit, Beijing Tiantan Hospital, Capital Medical University, Beijing, 100070 China

**Keywords:** Traumatic brain injury, Glasgow coma scale, Pupil reactivity, Arterial lactate concentration, Short term prognosis

## Abstract

**Background:**

The aim of the study was to determine whether the combination of Glasgow Coma Scale (GCS) and Pupil responses score (GCSP) with arterial lactate level would be an index to predict the short term prognosis in patients with traumatic brain injury (TBI).

**Methods:**

A retrospective study was performed enrolling all TBI patients admitted to intensive care unit (ICU) from 2019 to 2020. The demographics, clinical characteristics, and arterial lactate concentration were recorded. The GCSP and arterial blood analysis (ABG) with lactate was tested as soon as the patient was admitted to ICU. The Glasgow Outcome Scale (GOS) after discharge was regarded as the clinical outcome. A new index named GCSP-L was the combination of GCSP and lactate concentration. GCSP-L was the GCSP score (range 1-15) plus the lactate score (range 0-2). The lactate score was defined based on different lactate concentrations. If lactate was below 2 mmol/L, lactate score was 0, which above 5 mmol/L was 2 and between 2 and 5 mmol/L, the score was 1. As the range of GCSP was 1-15, the range of the GCSP-L was 1 to 17. The area under receiver operating characteristic curve (AUC) was calculated to evaluate the predictive ability of GCSP, lactate and GCSP-L. Statistical significance was set when *p* value < 0.05.

**Results:**

A total of 192 TBI patients were included in the study. Based on GCSP, mild, moderate, and severe TBI were 13.02, 14.06 and 72.92%, respectively. There were 103 (53.65%) patients with the lactate concentration below 2 mmol/L (1.23 ± 0.37 mmol/l), 63 (32.81%) of the range from 2 to 5 (3.04 ± 2.43 mmol/l) and 26 (13.54%) were above 5 mmol/l (7.70 ± 2.43 mmol/l). The AUC was 0.866 (95% CI 0.827-0.904) for GCSP-L, 0.812 (95% CI 0.765-0.858) for GCSP and 0.629 (95% CI 0.570—0.0.688) for lactate. The AUC of GCSP-L was higher than the other two, GCSP and lactate alone.

**Conclusions:**

The combination of GCSP and lactate concentration can be used to predict the short term prognosis in TBI patients.

## Background

Traumatic brain injury (TBI) is a common type of trauma worldwide [1] and a major cause of physical and cognitive disability [2]. It’s important for early identification of a patient’s clinical condition, reliable prognostic factors. Outcome prediction and severity evaluation are useful for clinical decision making, family counseling, evaluation of the quality of treatment, and medical resource allocation [1]. A simple combination of the Glasgow Coma Scale (GCS) and Pupil responses (GCSP) have been shown to be an early indicator of prognostic information and an extended index of clinical severity in TBI patients [3]. The GCSP is used to assess 4 aspects of a patient’s responsiveness, which are eye, verbal, motor responses, and pupil responses. Each of these aspects contains information about prognosis [4].

Nowadays the role of arterial lactate in TBI has gained great interest. Although higher arterial lactate may be a biomarker of poor systemic physiology, it may have neuroprotective effects on the intracranial pressure (ICP), the cerebral blood flow (CBF), and cerebral cellular metabolism [5, 6]. Some studies also have demonstrated that exogenously administered lactate has been associated with improved outcomes [7]. High lactate may be a protective factor for TBI patients. An arterial blood analysis (ABG) with lactate will be obtained immediately as soon as TBI patients are admitted to the intensive care unit (ICU). It’s rapid and convenient to have the arterial lactate concentration.

The GCSP score, together with the lactate concentration may be a simple and useful index to evaluate clinical severity and predict the prognosis. The aim of the study was to determine whether the combination of GCSP score with arterial lactate would be a good index to evaluate clinical severity and predict the prognosis in TBI patients.

## Methods

### Patients

This study was a retrospective study. The study was approved by the Ethics Committee of Beijing Tiantan Hospital. All TBI patients admitted to the ICU at Beijng Tiantan Hospital, Capital Medical University in China from 2019 to 2020 were enrolled in the study. Exclusion criteria: The patients with a history of chronic kidney and liver diseases, unstable hemodynamics, and obvious sign of infection (pulmonary, intracranial infection et al), were excluded. These diseases affect lactate concentration. And the patients with age under 18 years old, without ABG or Glasgow Outcome Scale (GOS) were excluded.

All the patients who met the criteria of TBI would be admitted directly to ICU of the hospital within 4 h of injury. All TBI patients received the same initial standardized treatment protocol, which included appropriate resuscitation and stabilization in accordance with the Seattle International Severe Traumatic Brain Injury Consensus Conference (SIBICC) [8]. After admitted to the ICU, neurologic evaluation will be performed immediately, including GCS score, pupil reactivity, and neurologic deficits. Patients were examined by CT scan as soon after stabilization as possible. According to CT findings, patients were taken either to the operating room for surgical evacuation of significant space-occupying lesions, or conservative management in ICU. CT scanning was repeated 6 and 24 hours after injury, as well as immediately after surgery, or when a patients level of consciousness deteriorated. An intracranial pressure (ICP) monitor or a ventricular catheter was inserted as needed. Arterial blood samples (ABG) were taken at admission for blood gas analysis.

### Data collection

The demographics and clinical characteristics were recorded after the patients were admitted to ICU, such as age, gender, body mass index (BMI), type of injury, acute physiology and chronic health evaluation II (APECHE II), injury severity score (ISS), initial GCS, pupil reactivity at admission of ICU. And every patient would have an ABG test for the first time after being admitted to ICU, thus the lactate concentration would be recorded.

ISS calculation divides the body into 6 areas. ISS is the sum of the squares of the highest abbreviated injury score (AIS) evaluation values of the 3 most severely injured areas. The ISS score ranges from 1 to 75 points. The mild to moderate is defined as ISS sore ranging from 1 to 8. And 9 to 15 is considered as serious injury, 16 to 24 is severe injury, ≥ 25 is critical injury.

GCSP is defined as the combination of GCS score and pupil reactivity score [3]. The pupil reactivity score is to reflect the number of nonreactive pupils. If both pupils are unreactive to light the score was 2. If only 1 pupil is unreactive to light the score is 1. If neither pupil is unreactive to light the score is 0. Thus, GCSP is obtained simply by subtracting the pupil reactivity score from the GCS total score. The range of GCSP is 1 to 15. GCSP score can be used to classify injuries, which 1 to 8 is mild, 9 to 12 is moderate and severe is range from 13 to 15 [3].

### The combination of GCSP and arterial lactate concentration (GCSP-L)

We first constructed a lactate score [9]. If the lactate concentration was below 2 mmol/L [9], the lactate score was 0. If it was above 5 mmol/L [6, 10], the score was 2. If it was between 2 and 5 mmol/L, the score was 1. Next, a combined GCSP-L was obtained by GCSP score plus the lactate score, GCSP-L = GCSP score + Lactate score. Since the GCSP score has a range from 1 to 15, the GCSP-L can range of possible values from 1 to 17.

### Outcome

Clinical outcome was assessed after discharge using the GOS. 1 = death, 2 = vegetative, 3 = severe disability, 4 = moderate disability, 5 = good recovery. Favorable outcomes were considered good recovery and moderate disability; severe disability, vegetative state, and dead were unfavorable. And ICU length of stay, hospital length of stay would be recorded.

### Statistical analysis

Given the nonnormality, data were expressed as mean with standard deviation (SD), and numbers and percentages for categorical variables. Receiver operating characteristic curve (ROC) analysis was used to estimate the prediction. The area under ROC (AUC) was calculated to evaluate the predictive ability of GCSP, lactate and GCSP-L. Statistical significance was set when *p* value < 0.05. All statistics were analyzed using GraphPad Prism version 8.0 (GraphPad Software, USA) and SPSS version 25.0 (IBM Corporation, USA).

## Results

**Fig. 1:**
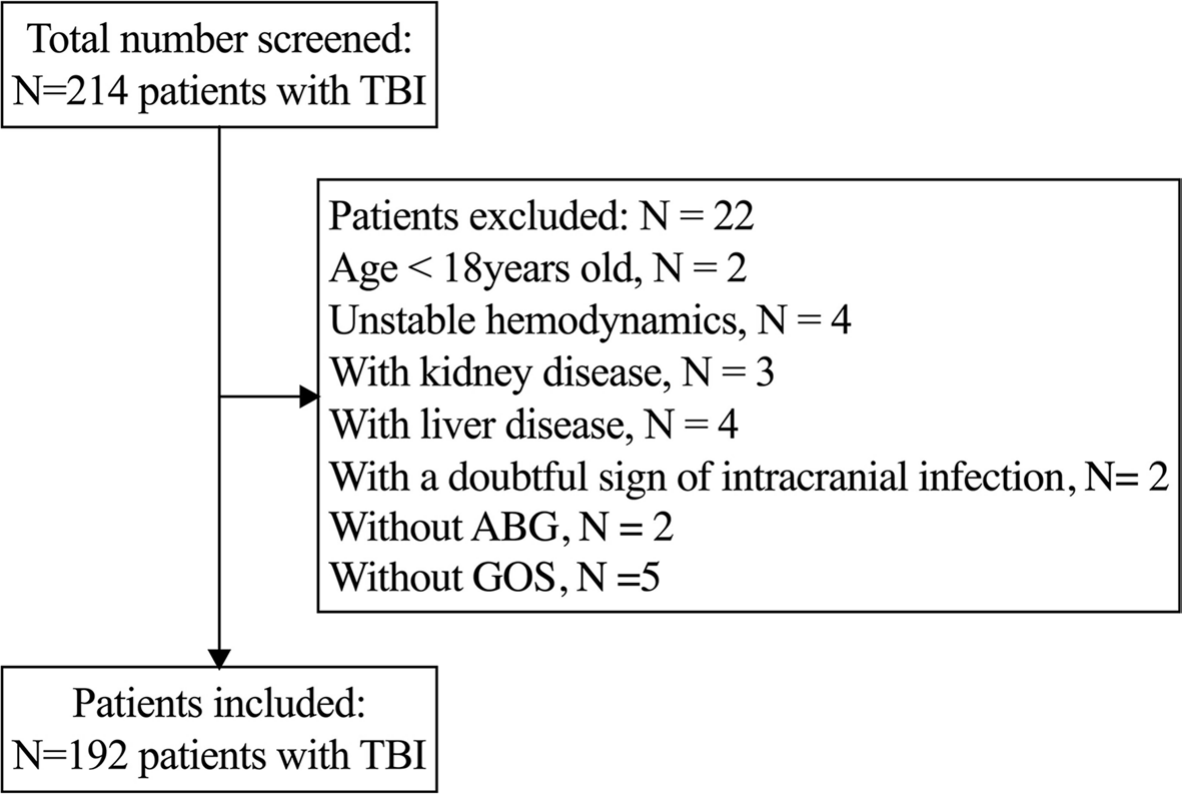
The flowchart of the study. A total of 214 patients with traumatic brain injury (TBI) were screened over the 2 years; 22 patients were excluded in the study. ABG = arterial blood analysis, GOS = Glasgow Outcome Scale

There were 214 TBI patients enrolled in the study. Out of 214 TBI patients, there were 2 patients without ABG after being admitted to ICU and 5 without GOS after discharge. Fifteen patients were excluded, 2 patients because they were below 18 years old, 3 because they have had kidney disease, 4 because of liver diseases, 4 for unstable hemodynamics, and 2 for a doubtful sign of intracranial infection. Thus, there were 192 TBI patients included in the study (Fig. 1). The TBI patients would be admitted to ICU. All the patients were treated in accordance with the standardized management protocol [8].

### Demographics, admission status and clinical outcome

Of the 192 TBI patients included in the study, the mean age was 54 ± 16 years old and 75% were male (Table 1). The types of TBI were intracranial hemorrhage (ICH), subdural hemorrhage (SDH), epidural hemorrhage (EDH), diffuse axonal injury, and others, respectively 51.56, 27.08, 14.06, 4.69, and 2.60%. The critical patients were 69.79% among the patients according to ISS (Table 1). Head AIS made the maximal contribution in ISS score of all the patients. According to CT findings, 112 patients (58.33%) were taken to the operating room for surgery. Of all TBI patients, 65.63% (126 patients) were received mechanical ventilation. Most of the patients were received sedation and analgesia, 95.83 and 96.88% respectively.

**Table 1:** Demographics, admission status and clinical outcome of TBI Patients

Variable	Value
Male (n [%])	144 ± (75%)
Age (year)	54.14 ± 16.94
Height (cm)	168.76 ± 8.31
Weight (kg)	69.99 ± 12.21
BMI (kg/m^2^)	24.48 ± 3.32
Type of TBI	
Intracranial hemorrhage (ICH)	99 (51.56%)
Subdural hemorrhage (SDH)	52 (27.08%)
Epidural hemorrhage (EDH)	27 (14.06%)
Diffuse axonal injury	9 (4.69%)
Others	5 (2.60%)
ISS score	
Mild to moderate (1–8) (n [%])	3 (1.56%)
Head AIS (M,IOQ)	2 (1.5, 2)
Sum of non-head AIS (M,IOQ)	1 (0.5,1.5)
Serious (9–15) (n [%])	19 (9.90%)
Head AIS	3 (3, 3)
Sum of non-head AIS (M,IOQ)	1 (1, 1)
Severe (16–24) (n [%])	36 (18.75%)
Head AIS	4 (4, 4)
Sum of non-head AIS (M,IOQ)	1 (0, 1)
Critical (25–75) (n [%])	134 (69.79%)
Head AIS	5 (5, 5)
Sum of non-head AIS (M,IOQ)	1 (1, 1)
GCSP score	
Mild (13–15) (n [%])	25 (13.02%)
Moderate (9–12) (n [%])	27 (14.06%)
Severe (1–8) (n [%])	140 (72.92%)
Surgery (n [%])	112 (58.33%)
APACHE II	43.8 ± 12.74
Mechanical ventilation (n [%])	126 (65.63%)
Analgesia (n [%])	186 (96.88%)
Sedation (n [%])	184 (95.83%)
ICU length of stay (days)	19 ± 18
GOS after discharge	
1 (death) (n [%])	36 (18.75%)
2 (vegetative) (n [%])	11 (5.73%)
3 (severe disability) (n [%])	92 (47.92%)
4 (moderate disability) (n [%])	23 (11.98%)
5 (good recovery) (n [%])	30 (15.63%)
Hospital length of stay (days)	23 ± 21
Lactate concentration (mmol/L)	
< 2 (Mean ± SD [n(%)])	1.23 ± 0.37 [103 (53.65%)]
2 - 5 (Mean ± SD [n(%)])	3.04 ± 2.43 [63 (32.81%)]
> 5(Mean ± SD [n(%)])	7.70 ± 2.43 [26 (13.54%)]

*BMI* Body mass index, *TBI* Traumatic brain injury, *ISS* Injury Severity Score, *GCS-P* Glasgow Coma Scale and Pupil response score, *APACHE II* Acute physiology and chronic health evaluation II, *ICU* Intensive care unit, *GOC* Glasgow outcome score, *AIS* Abbreviated Injury Score, *M(IQR)* Median (Interquartile range)

Based on GCSP, the score between 13 to 15 considered as mild was 13.02%, moderate (9-12) and severe (1-8) were 14.06 and 72.92%, respectively. The percentage of favorable outcomes was 27.62% (GOS after discharge: good recovery 15.63%, moderate disability 11.98%). ICU length of stay was 19 ± 18 days, and hospital length of stay (23 ± 21) days (Table 1).

There were 103 (53.65%) patients with the lactate concentration below 2 mmol/L (1.23 ± 0.37 mmol/l). Meanwhile, the number of range from 2 to 5 was 63 (32.81%), and the mean lactate was 3.04 ± 2.43 mmol/l. The others (26 (13.54%)) were above 5 mmol/l, and mean lactate was 7.70 ± 2.43 mmol/l (Table 1).

### Sensitivity and specificity based on GCSP, GCSP-L and lactate

**Fig. 2:**
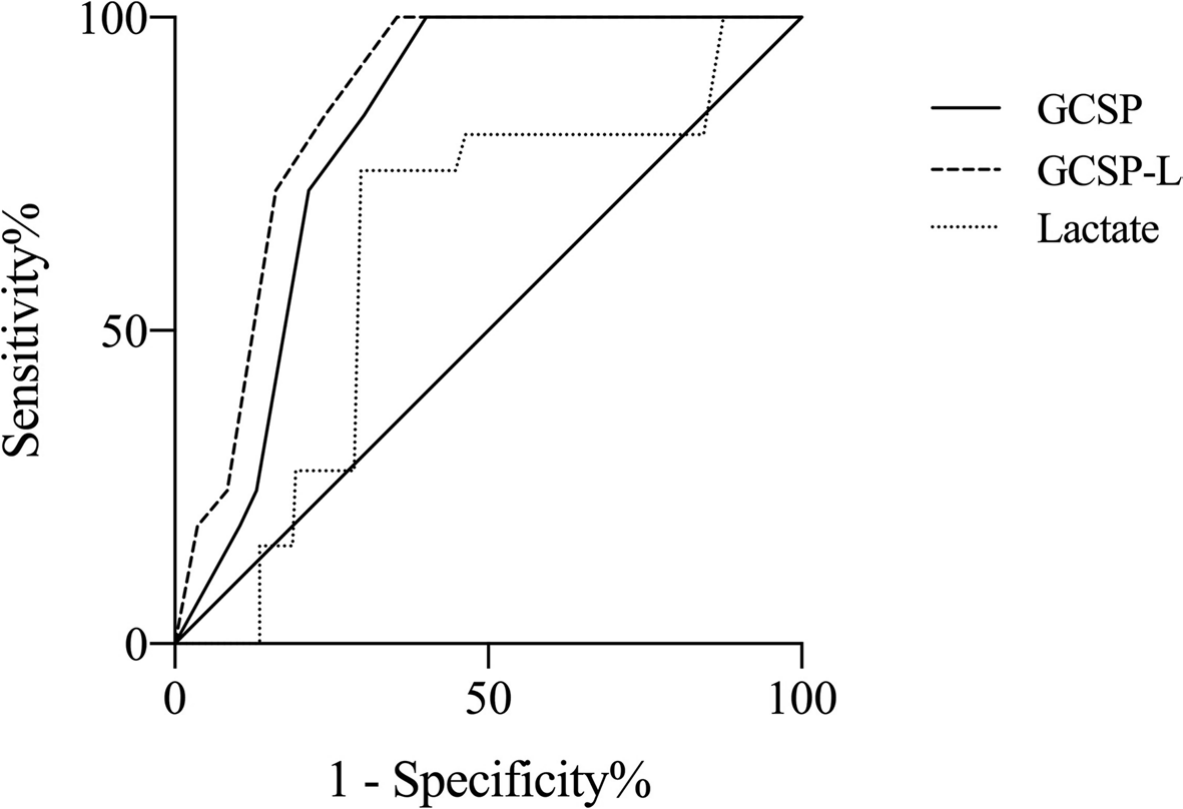
The receiver operating curves (ROC) of GCSP, lactate and GCSP-L. The area under the receiver operating characteristic curve (AUC) was 0.866 (95% CI 0.827-0.904) for GCSP-L, 0.812 (95% CI 0.765-0.858) for GCSP and 0.629 (95% CI 0.570—0.0.688) for lactate. GCSP = Glasgow Coma Scale and Pupil response score, GCSP-L = Glasgow Coma Scale, Pupil response and Lactate score

The ROC curves are shown in Fig. 2. The GCSP-L achieved the largest AUC of 0.866 (95% confidence interval [CI], 0.827-0.904), which was higher than the others, 0.812 (95% CI 0.765-0.858) for GCSP and 0.629 (95% CI 0.570—0.0.688) lactate respectively.

## Discussion

Lactate is an important biomarker of systemic physiology and may have neuroprotective effects in TBI patients. GCS score and pupil reaction are two clinical features, that provide some information about prognosis for a brain-injuryed patient. This study combined the lactate concentration and GCSP score, which named GCSP-L, to predict short term prognosis in TBI patients. The findings highlighted that GCSP-L can be used to predict the short term prognosis, and may provide more information than either GCSP or lactate alone.

GCS score is widely used as an index of brain damage, differing severity and monitoring patients’ progress and estimating prognosis [11]. GCS score assess 3 aspects of a patient’s responsiveness, they are eye, verbal, and motor responses [4]. Each of these aspects contains information about prognosis. Recently, a simple combination of GCS and Pupil responses (GCSP) have been shown to be an early indicator of prognostic information and an extended index of clinical severity in TBI patients [3]. GCSP yields more informative data than using either GCS or pupil reaction alone. Nowadays, some researches are using GCSP to prognosis and estimate severity in TBI patients [12]. So this study used GCSP as the reference index to estimate severity and prognosis.

High arterial lactate is considered to be a prognosis factor of poor systemic physiology and poor outcome, however, in TBI patients it may have a protective and beneficial effect [6]. A study demonstrated that there was a negative correlation between arterial lactate and ICP in severe TBI patients [13]. ICP decreased while arterial lactate increased. High arterial lactate could regulate the cerebral vessels by vasodilation leading to reduce ICP [7] and increases in the CBF [5]. With these effects, high lactate may be beneficial for TBI patients. Further the role of arterial lactate as cerebral energy fuel in TBI has gained interest the last years [14, 15]. Some researches have showed that exogenous supplemental lactate can be utilized aerobically as a preferential energy substrate after TBI [7]. The experimental group gave an intervention of a 3-h intravenous infusion of hypertonic sodium lactate (aiming to increase systemic lactate to 5 mmol/L). With the sodium lactate therapy, a significant increase in cerebral microdialysis concentrations of lactate was observed and also a decrease of ICP. In a word, high lactate may have neuroprotective effects and beneficial effects at TBI patients. Thus, the study used GCSP plus lactate score as a new index (GCSP-L) to evaluate the severity of TBI patients.

Generally, hyperlactatemia is defined as lactate levels > 2 mmol/L, whereas lactic > 4 mmol/L represents acidosis [9]. But in this study we used 5 mmol/l as the second grading criteria. If it was above 5 mmol/L, the score was 2. If it was between 2 and 5 mmol/L, the score was 1. Because some studies demonstrated that the arterial lactate contribution to cerebral lactate will increase to 60% at 5 mmol/L [6, 10], which will serve as cerebral energy fuel especially the cerebral lactate. Some studies showed that infusing exogenous supplemental lactate to maintain lactate concentration above 5 mmol/l, the lactate can be utilized as cerebral energy fuel [7].

After admitted to the hospital or ICU, GCSP assessment and a ABG test will be performed immediately. It’s very easy and rapid to have the results of the combination of GCSP and lactate concentration. Although GCSP is widely used for assessment of a patient’s clinical condition, the AUC of GCSP-L is greater than GCSP and lactate (AUC 0.866, 0.812 and 0.629 respectively). GCSP-L can be used to predict the short term prognosis and GCSP-L may provide more information about clinical severity and outcomes than either GCSP or lactate alone.

There are some limitations in the study. First, the study screened all the TBI patients immediately after admitted to the ICU within 4 hours. But we didn’t have the exact data of the time from the patients were injured to visiting the hospital. If the lactate can be obtained at once TBI happened, the GCSP-L may be more precise to evaluate the short term prognosis. Second, there were 69.79% critical patients according to ISS. The patients were severe and poor prognosis. However, the patients with severe unstable hemodynamic were excluded. Third, this was a retrospective study. A larger patient population and randomized controlled trials should be performed.

## Conclusions

The combination of GCSP and lactate concentration can be used to predict short term prognosis in TBI patients.

## Data Availability

The datasets analysed during the current study are available from the corresponding author on reasonable request.

## Abbreviations

TBI: Traumatic Brain Injury
GCS: Glasgow Coma Scale
GCSP: Glasgow Coma Scale and Pupil responses
ICP: Intracranial Pressure
CBF: Cerebral Blood Flow
ABG: An Arterial Blood Analysis
ICU: Intensive Care Unit
GOS: Glasgow Outcome Scale
SIBICC: the Seattle International Severe Traumatic Brain Injury Consensus Conference
BMI: Body Mass Index
APECHE II: Acute Physiology and Chronic Health Evaluation II
AIS: Highest Ais Evaluation
ISS: Injury Severity Score
GCSP-L: Glasgow Coma Scale, Pupil responses and Arterial Lactate Concentration
ROC: Receiver Operating Characteristic Curve
AUC: the Area under Receiver Operating Characteristic Curve
ICH: Intracranial Hemorrhage
SDH: Subdural Hemorrhage
EDH: Epidural Hemorrhage
